# Risk Factors for Carbapenem-Resistant *Klebsiella pneumoniae* Infection: A Meta-Analysis

**DOI:** 10.1089/mdr.2017.0061

**Published:** 2018-03-01

**Authors:** Pin Liu, Xuan Li, Mei Luo, Xuan Xu, Kewen Su, Shuai Chen, Ying Qing, Yingli Li, Jingfu Qiu

**Affiliations:** School of Public Health and Management, Chongqing Medical University, Chongqing, China.

**Keywords:** carbapenem-resistant *Klebsiella pneumoniae*, infection, risk factor, meta-analysis

## Abstract

***Aims:*** Carbapenem-resistant *Klebsiella pneumoniae* (CRKP) infection has been rapidly emerging as a life-threatening nosocomial disease in many countries. However, studies on the corresponding risk factors of CRKP infection showed inconsistent results. To resolve these inconsistencies, we conducted a meta-analysis of previous studies on the potential risk factors of CRKP infection. The results of this study could be used to develop CRKP infection prevention strategies.

***Methods:*** Relevant works were systematically searched from five electronic databases up to September 2016. *Z*-test was used to determine the significance of the pooled odds ratios (ORs). ORs and 95% confidence intervals were utilized to compare the risk factors of CRKP infection.

***Results:*** Sixteen studies that involved 3,627 participants were included in the meta-analysis. We identified the following risk factors that were associated with CRKP infection: (1) longer length of hospital stay (LOS) (OR = 12.92), (2) admission to intensive care unit (ICU) (OR = 2.48), (3) prior hospitalization (OR = 1.85), (4) longer days of ICU stay (OR = 4.58), (5) transplant recipient (OR = 2.01), (6) steroid use (OR = 1.43), (7) central venous catheter use (OR = 2.30), (8) mechanical ventilation (OR = 2.54), (9) presence of tracheostomy (OR = 3.63), (10) parenteral nutrition (OR = 2.38), (11) previous antibiotic use (OR = 3.31), and (12) exposure to carbapenems (OR = 4.01), (13) aminoglycosides (OR = 2.05), (14) glycopeptides (OR = 2.40), (15) quinolones (OR = 2.28), and (16) anti-pseudomonal penicillins (OR = 2.67).

***Conclusions:*** Sixteen risk factors including longer LOS, admission to ICU, previous antibiotic use, and exposure to carbapenems were associated with the development of CRKP infection. Identification of modifiable risk factors could play an important role in the prevention of CRKP infection.

## Introduction

Carbapenems are the first-line therapy for infections caused by multidrug-resistant Gram-negative Enterobacteriaceae, especially extended-spectrum β-lactamase-producers.^1^ In the recent years, however, the emergence of carbapenem-resistant Enterobacteriaceae (CRE) strains has been increasingly reported.^2^ CRE strains provide a particular challenge because they are resistant to β-lactam agents and there are very limited treatment options for CRE induced diseases. Moreover, these bacterial strains show the potential to spread within healthcare facilities. Infections caused by CRE are associated with high morbidity and mortality rates. In a meta-analysis of deaths attributable to CRE infections, CRE-attributable deaths varied from 26% to 44%.^3,4^

*Klebsiella pneumoniae* is one of the most common Enterobacteriaceae that causes nosocomial infections, such as septicemia, pneumonia, and urinary tract infection, surgical site infection, and catheter-related infection.^5^ Among CRE species, carbapenem-resistant *K. pneumoniae* (CRKP) poses a major threat, causing an alarming increase in infection rate over the last years. It has been reported that from 2005 to 2011, the proportion of *K. pneumoniae* isolates resistant to carbapenems increased from 28% to 68.2% in Greece.^6,7^

Patients infected with CRKP are often chronically and acutely ill, which is associated with high mortality. The mortality of patients with CRKP infection (main blood infection) was up to 70%,^8^ another study reported that 14-day mortality of 19 patients with bacteremia due to *Klebsiella pneumoniae* carbapenemases (KPC)-producing *K. pneumoniae* was 47%.^9^ Moreover, the readmission rate of survivors was approximately 72% within 90 days of discharge.^10^ Infections with such strains are difficult to control, because carbapenemase-resistant genes can potentially spread within and between hospitals via transferable plasmids.^11^ Therefore, knowledge of risk factors associated with CRKP infection development is important to identify high-risk patients in the prevention of CRKP acquisition. In addition, such knowledge also is essential in the empirical therapeutic decision-making process and in the design of effective control measures to prevent infection.

Several studies have evaluated the risk factors of CRKP infection, but their results remain controversial.^12–15^ For example, one study reported that treatment with quinolones is a risk factor of CRKP infection,^16^ whereas other studies showed no association between the use of quinolones and CRKP infection.^13,14^ Thus, a meta-analysis is needed to resolve these inconsistent results, because a well-designed and appropriately performed meta-analysis can become a powerful analytical method where both independent and different studies are integrated and their results are pooled, thereby increasing the power of statistical testing and producing information that cannot be drawn from one individual study.^17^ Accordingly, we performed a meta-analysis to identify the relationship between risk factors and CRKP infection.

## Materials and Methods

A meta-analysis of observational studies on risk factors of CRKP infection was conducted according to the Preferred Reporting Items for Systematic Reviews and Meta-Analyses (PRISMA) guidelines.^18^ The methodology included data source collection, inclusion and exclusion criteria, data extraction, quality assessment, and statistical analysis.

### Data source collection and screening strategy

A systematic search of English written articles up to September 2016 that focused on the risk factors of CRKP infection was performed on PubMed/Medline, Embase, Web of Science, Cochrane Central Register of Controlled Trials, and EBSCO. The following search terms were used: (carbapenem-resistant or carbapenemase-producing or KPC) and (*Klebsiella pneumoniae* [MeSH] or *Klebsiella pneumoniae*) and (infection [MeSH] or infections or infection) and (risk factors [MeSH] or factor, risk or factors, risk or risk factor or dangerous factors or hazards or causes).The references to all identified published works were entered into a reference management software program (EndNote, version X7; Thomson Reuters, Toronto, ON, Canada).

### Inclusion and exclusion criteria

Two reviewers (P.L. and X.L.) conducted an initial screening of titles and abstracts independently. A second screening was completed through a full-text review by the same reviewers. Subsequently, we compared the screened studies to determine whether they were in accordance with the cross-check method. Any disagreements were addressed by a third party (J.Q.) when necessary.

The inclusion criteria were as follows: (1) studies were about the risk factors for CRKP infection. (2) CRKP was defined as the resistance of *K. pneumoniae* to imipenem, meropenem, or ertapenem based on the susceptibility breakpoints that had been applied by the investigators of each study, which were identified through definite microbiological methods (Vitek automated system, BD Phoenix automated microbiology system, modified Hodge test, disc diffusion method, and E-test), and the definition of infection was explicit. (3) It was a case–control or cohort study in design. (4) The studies were published in English.

Studies were excluded if they were (1) duplicated studies, (2) reviews, reports, or meeting abstracts, (3) studies that did not distinguish the outcomes of infected patients from those of colonized patients, (4) studies that did not provide sufficient information to allow the calculation of odds ratios (ORs) and 95% confidence intervals (CIs).

### Data extraction

Three reviewers (P.L., X.L., and K.S.) independently extracted the relevant data according to a previously created data extraction form. The extracted data included (1) the title of studies and years of publication, (2) the first author's name and country where the study was implemented, (3) study designs, (4) number of cases and control patients, and (5) all identified risk factors of CRKP infection and ORs of risk factors calculated from both univariate and multivariate logistic regression analyses. The extraction results were evaluated by other reviewers (Y.L. and M.L.), and any disagreements were resolved through discussion.

### Quality assessment

The quality of each study was assessed based on the nine-star Newcastle–Ottawa Scale (NOS),^19^ which included three aspects of methodology assessment: selection of cases (4 items, 4 points), comparability of cases and controls (1 item, 2 points), and ascertainment of exposure to risk factors (3 items, 3 points). Scores of 0–4 points indicated a low-quality research, whereas scores of 5–9 points suggested a high-quality research.^20^ Three reviewers (S.C., Y.Q., and X.X.) independently assessed the quality of each study, and different opinions on scoring were resolved through discussion among the research group until a consensus was reached.

### Statistical analysis

The meta-analysis was performed using Review Manager (RevMan) software version 5.3 (The Cochrane Collaboration, Copenhagen, Denmark) and Stata version 11.0 (StataCorp, College Station, TX). The *χ*^2^ and *I*^2^ statistic tests were used to evaluate the heterogeneity among the included studies. The random-effects model was utilized to combine the results when heterogeneity was present among the studies (*I*^2^ *>* 50% or *p* < 0.05). Otherwise, the fixed-effects model would be used. Pooled ORs with a 95% CI were calculated using either a fixed-effects or a random-effects model to compare the risk factors of CRKP infection. The *Z*-test was used to determine the significance of the pooled ORs. The results were considered statistically significant when *p* < 0.05. Sensitivity analyses were conducted through sequential omission of individual studies and then comparison of the *p* value of pooled ORs. The results were identified as credible when the corresponding *p* value of pooled ORs was not substantially different. Potential publication bias was examined using Egger's test through the software Stata version 11.0. Results were considered statistically significant when *p* < 0.05. In addition, the overall population exposure rate (*P_e_*) was replaced by the pool exposure rate of controls to calculate the population attributable risk proportion (PARP) following the formula: PARP = *P_e_* (OR −1)/[*P_e_* (OR −1) + 1].

## Results

### Study selection and characteristics

**FIG. 1.**
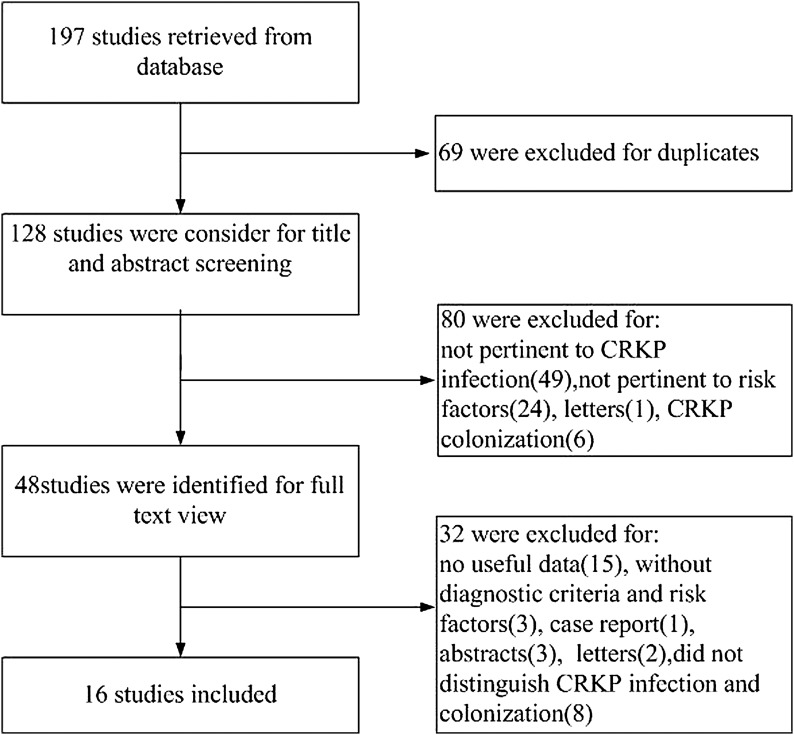
Preferred reporting items for systematic reviews and meta-analysis flowchart of the study identification and selection. CRKP, carbapenem-resistant *Klebsiella pneumoniae.*

Following the literature search and selection, 197 potentially relevant publications up to September 2016 were systematically retrieved from the electronic databases. After the title and abstract reviews, 48 studies were retained, and duplicates, letters, meeting abstracts, case reports, and studies not pertinent to the risk factors of CRKP infection were excluded. Among them, 32 studies that did not match the inclusion criteria were excluded via full-text screening. Finally, 16 studies^13,14,16,21–33^ were included in the meta-analysis (Fig. 1). Table 1 lists the detailed characteristics of the 16 included articles.

**Table 1.** Characteristics of Studies Included in the Meta-Analysis

*Study*	*Study design*	*Period*	*Study location*	*Case/control, n*	*Mean age (SD or range), case/control, years*	*Male sex, case/control,* n	*Quality assessment*^a^	*Adjusted variables*
Falagas, 2007	Case-control	October 2000–May 2006	Greece	53/53	61.5 (18.8)/61.9 (17.2)	38/39	6 points	Fluoroquinolones, antipseudomonal penicillins
Patel, 2008	Case-control	July 2004–June 2006	USA	99/99	60.67 (14.95)/59.39 (13.34)	58/58	7 points	Liver disease, renal insufficiency, transplant recipient, receipt of MV, use of CVC, ICU stay, length of stay before infection, cephalosporin, fluoroquinolone, carbapenem, β-lactam and/or β-lactamase inhibitor, monobactam, aminoglycoside
Wu, 2011	Case-control	July 2006–July 2008	China	39/78	64 (16.0)/56.9 (17.6)	28/60	7 points	ICU admission (within 2 weeks), carbapenems, lycopeptides
Borer, 2012	Case-control	May 2007–January 2010	Israel	42/84	72 (19–91)/72.5 (21–95)^b^	NA	6 points	Previous invasive procedures, diabetes mellitus, solid tumor, tracheostomy, urinary catheter insertion, antipseudomonal penicillin use
Correa, 2013	Case-control	January 2006–August 2008	Brazil	20/40	59.6/64.9	13/21	7 points	Mean APACHE II score at admission, CVC, mean urinary catheter use, prior antimicrobial use
Gómez, 2014	Case-control	January 2008–January 2011	Colombia	61/122	42.2 (28.4)/47.3 (24.3)	30/75	8 points	Exposure to quinolones, time at risk, charlson index, colonization, enteral nutrition, carbapenems, piperacillin-tazobactam
Simkins, 2014	Case-control	January 2006–December 2010	USA	13/39	53 (18)/55 (16)	7/14	5 points	NA
Hu, 2016	Case-control	January 2011–June 2013	China	65/65	64.12 (13.69)/59.06 (14.61)	45/50	6 points	Age, prior hospitalization, number of antibiotic groups, carbapenems
Giannella, 2014	Case-control	January 2012–December 2013	Italy	143/752	65 (52–75)/70 (58–81)^b^	84/307	6 points	Admission to ICU, invasive abdominal procedures, chemotherapy/radiation therapy, colonization at site besides stool
Mouloudi, 2014	Cohort	January 2008–December 2011	Greece	17/34	54 (44–66)/55 (26–44)^b^	10/19	5 points	NA
Brizendine, 2015	Cohort	2006–2012	USA	22/64	56 (10.3)/51 (12.8)	16/14	7 points	NA
Candevir, 2015	Cohort	January 2012–December 2012	Turkey	47/51	38 (0–83)/8 (0–86)^b^	31/30	7 points	Third-generation cephalosporin, nasogastric catheter use, Being admitted to neurosurgical ICU
Freire, 2015	Cohort	January 2009–December 2013	Brazil	25/1,076	59 (21–73)/47 (6–79)^b^	14/571	6 points	Age, double transplant, ureteral stent
Giannella, 2015	Cohort	June 2010–December 2013	Italy	20/217	63 (2.8)/55 (14)	15/143	7 points	Renal replacement therapy, MV >48 hr, histological recurrence of HCV, CRKP rectal carriage at any time
Ny, 2015	Cohort	January 2011–December 2013	USA	48/48	70 (36–95)/78 (29–95)^b^	23/25	7 points	NA
Vardakas, 2015	Cohort	January 2006–October 2009	Greece	73/18	66.3 (14.4)/60.9 (15.6)	36/7	7 points	NA

^a^ Low-quality research, 0–4 points; high-quality research, 5–9 points.

^b^ Age, median (range), years.

APACHE, Acute Physiology and Chronic Health Evaluation; CRKP, carbapenem-resistant *Klebsiella pneumoniae*; CVC, central venous catheter; HCV, hepatitis C virus; ICU, intensive care unit; MV, mechanical ventilation; NA, not available; SD, standard deviation.

A total of 3,627 participants (787 cases and 2,840 controls) were contained in this meta-analysis, and all participants were inpatients. The sample sizes in studies ranged from 51 to 1,101. These included studies were published from 2007 to 2016 in different countries including the United States,^21,23,27,31^ China,^14,24^ Brazil,^29,33^ Italy,^25,30^ Israel,^22^ Colombia,^13^ Turkey,^28^ and Greece.^16,26,32^ Of the 16 studies, 6 studies were retrospective cohort study designs,^26–29,31,32^ 1 study was a prospective cohort study design,^30^ and the other 9 studies were case–control designs.^13,14,16,21–25,33^ Among those studies, the results of four cohort studies^26,27,31,32^ and one case–control study^23^ did not adjust for any potential confounders, whereas the remaining studies adjusted for several conventional risk factors. All studies were considered as high-quality research after assessment via the NOS.

### Risk factors of CRKP infection

**FIG. 2.**
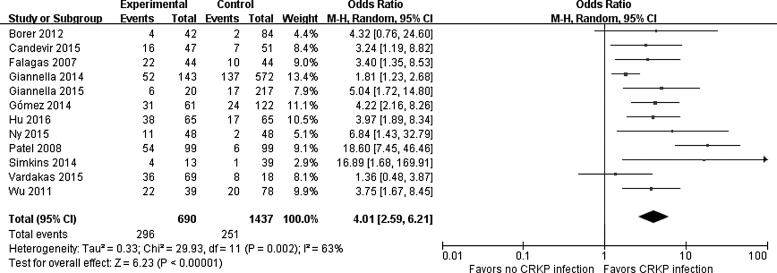
Forest plot for the association between exposure to carbapenems and CRKP infection. The individual block squares denote the mean difference for each study of the risk factor exposure to carbapenems, with an area proportional to the amount of statistical information in each study. The *horizontal line* denotes 95% CI. The pooled estimate and its 95% CI are represented by a *diamond*. CI, confidence interval; M-H, Mantel-Haenszel.

Table 2 shows the risk factors of CRKP infection and heterogeneity in the meta-analysis. Among the risk factors, length of hospital stay (LOS), days of intensive care unit (ICU) stay, Acute Physiology and Chronic Health Evaluation (APACHE) II score on ICU, and charlson comorbidity index were continuous variables, the rest were binary variables. The size of statistical heterogeneity among the studies was evaluated using *I*^2^ statistics.^34^ A significant relationship was found between CRKP infections and the following risk factors: longer LOS (OR = 12.92; 95% CI = [6.84–19.00]), admission to ICU (OR = 2.48; 95% CI = [1.90–3.23]), prior hospitalization (OR = 1.85; 95% CI = [1.12–3.07]), longer days of ICU stay (OR = 4.58; 95% CI = [3.67–5.49]), transplant recipient (OR = 2.01; 95% CI = [1.03–3.92]), steroids use (OR = 1.43; 95% CI = [1.04–1.96]), central venous catheter (CVC) use (OR = 2.30; 95% CI = [1.26–4.19]), mechanical ventilation (MV) (OR = 2.54 95% CI = [1.67–3.85]), presence of tracheostomy (OR = 3.63; 95% CI = [1.47–9.00]), and parenteral nutrition use (OR = 2.38; 95% CI = [1.68–3.36]); previous antibiotic use (OR = 3.31; 95% CI = [1.68–6.49]); carbapenems (OR = 4.01; 95% CI = [2.59–6.21]), aminoglycosides (OR = 2.05; 95% CI = [1.43–2.94]), glycopeptides (OR = 2.40; 95% CI = [1.09–5.27]), quinolones (OR = 2.28; 95% CI = [1.40–3.70]), and anti-pseudomonal penicillins (OR = 2.67; 95% CI = [1.78–4.01]). Figure 2 illustrates a forest plot describing the relationship between carbapenems exposure and CRKP infection.

**Table 2.** Meta-Analysis of Risk Factors of Carbapenem-Resistant *Klebsiella pneumoniae* Infection

						*Heterogeneity of study design*				
*Risk factor*	*Combination studies*	*Case/controls*	*OR/MD [95% CI]*	Z	p	*χ^2^*	p	I^*2*^*(%)*	*Analysis model*	*Egger's test (*p*)*
Prior hospitalization	3	131/157	1.85 [1.12–3.07]	2.38	0.02^*^	0.00	1.00	0	Fixed	0.374
LOS	10	592/1,402	12.92 [6.84–19.00]^a^	4.16	0.000^*^	36.90	0.000	76	Random	0.356
Admission to ICU	8	466/1,215	2.48 [1.90–3.23]	6.69	0.000^*^	14.04	0.05	50	Fixed	0.219
Days of ICU stay	4	193/455	4.58 [3.67–5.49]^a^	9.87	0.000^*^	3.62	0.31	17	Fixed	0.214
APACHE II score on ICU admission	4	208/170	0.25 [−0.98–1.47]^a^	0.40	0.69	2.61	0.460	0	Fixed	0.870
Comorbidity										
DM	10	473/1,597	1.31 [0.97–1.78]	1.78	0.08	9.21	0.42	2	Fixed	0.441
Malignancy	5	347/819	0.72 [0.47–1.11]	1.48	0.14	1.48	0.83	0	Fixed	0.208
Heart disorders	4	234/251	1.28 [0.85–1.92]	1.20	0.23	3.71	0.29	19	Fixed	0.894
Transplant recipient	5	348/1,909	2.01 [1.03–3.92]	2.04	0.04^*^	11.27	0.02	65	Random	0.993
Renal failure	3	165/169	1.68 [0.83–3.41]	1.43	0.15	1.72	0.42	0	Fixed	0.314
Steroid use	5	328/877	1.43 [1.04–1.96]	2.23	0.03^*^	2.13	0.71	0	Fixed	0.486
Respiratory disease	3	129/130	1.38 [0.72–2.66]	0.98	0.33	0.63	0.73	0	Fixed	0.844
Liver disease	3	225/170	1.45 [0.86–2.44]	1.39	0.17	0.95	0.62	0	Fixed	0.890
Neurological disorders	3	190/136	1.43 [0.87–2.35]	1.40	0.16	0.33	0.85	0	Fixed	0.554
Charlson comorbidity index	3	252/742	−0.89 [−2.31–0.53]^a^	1.23	0.22	41.18	0.000	95	Random	0.290
Invasive procedures										
CVC use	8	538/1,039	2.30 [1.26–4.19]	2.71	0.007^*^	28.23	0.000	79	Random	0.344
MV	9	577/1,117	2.54 [1.67–3.85]	4.37	0.000^*^	20.15	0.010	60	Random	0.739
Prior surgery	7	456/953	1.45 [0.89–2.36]	1.47	0.14	17.37	0.008	65	Random	0.195
Urinary catheter	7	388/986	1.60 [0.86–2.98]	1.50	1.13	28.71	0.000	79	Random	0.305
Tracheostomy	5	243/385	3.63 [1.47–9.00]	2.79	0.005^*^	13.73	0.008	71	Random	0.036
Parenteral nutrition	3	275/712	2.38 [1.68–3.36]	4.90	0.000^*^	2.27	0.320	12	Fixed	0.852
Previous antibiotic use	10	569/1,295	3.31 [1.68–6.49]	3.47	0.000^*^	32.25	0.000	72	Random	0.094
Antibiotic										
Carbapenems	12	690/1,437	4.01 [2.59–6.21]	6.23	0.000^*^	29.93	0.002	63	Random	0.142
β-lactam and/or β-lactamase inhibitor	4	320/775	3.56 [0.85–14.87]	1.74	0.08	51.82	0.000	94	Random	0.509
Cephalosporins	8	533/1,037	1.64 [0.90–2.98]	1.63	0.10	27.69	0.000	75	Random	0.734
Aminolycosides	8	533/1,037	2.05 [1.43–2.94]	3.91	0.000^*^	8.75	0.27	20	Fixed	0.366
Glycopeptides	3	226/694	2.40 [1.09–5.27]	2.18	0.03^*^	6.41	0.04	69	Random	0.326
Quinolones	10	601/1,302	2.28 [1.40–3.70]	3.32	0.000^*^	26.66	0.002	66	Random	0.101
Metronidazole	3	184/244	0.68 [0.14–3.29]	0.48	0.63	11.43	0.003	82	Random	0.890
Anti-pseudomonal penicillins	5	263/319	2.67 [1.78–4.01]	4.73	0.000^*^	2.01	0.73	0	Fixed	0.495

^a^ Mean difference.

^*^ *p* < 0.05.

CI, confidence interval; DM, diabetes mellitus; LOS, length of hospital stay; MD, mean difference; OR, odds ratio.

### PARP of risk factors

The PARP of risk factors for CRKP infection was further carried out. The calculation of PARP can help us to learn the main risk factors of CRKP infection. Table 3 shows that the PARP of previous antibiotic use was up to 62.42%. In addition, other variables, such as admission to ICU (PARP = 25.03%); prior hospitalization (PARP = 27.48%); use of CVC (PARP = 40.37%), MV (PARP = 34.56%), presence of tracheostomy (PARP = 25.08%), and parenteral nutrition (PARP = 19.12%); carbapenems (PARP = 34.46%), quinolones (PARP = 23.87%), and anti-pseudomonal penicillins (PARP = 24.80%) exposure were also high-risk factors for CRKP infection. The risk factors LOS, days of ICU stay are not displayed in Table 3 because the original literatures did not provide sufficient information on the calculation of the *P_e_* value.

**Table 3.** The Population Attributable Risk Proportion of Risk Factors for Carbapenem-Resistant *K. pneumoniae* Infection

Risk factor	OR [95% CI]	P_e_*(%)*	PARP (%)
Previous antibiotic use	3.31 [1.68–6.49]	71.89	62.42
CVC use	2.30 [1.26–4.19]	52.07	40.37
MV	2.54 [1.67–3.85]	34.29	34.56
Carbapenems	4.01 [2.59–6.21]	17.47	34.46
Prior hospitalization	1.85 [1.12–3.07]	44.59	27.48
Tracheostomy	3.63 [1.47–9.00]	12.73	25.08
Admission to ICU	2.48 [1.90–3.23]	22.56	25.03
Anti-pseudomonal penicillins	2.67 [1.78–4.01]	19.75	24.80
Quinolones	2.28 [1.40–3.70]	24.50	23.87
Parenteral nutrition	2.38 [1.68–3.36]	17.13	19.12
Glycopeptides	2.40 [1.09–5.27]	15.71	18.03
Steroid use	1.43 [1.04–1.96]	22.35	8.77
Transplant recipient	2.01 [1.03–3.92]	8.49	7.90
Aminoglycosides	2.05 [1.43–2.94]	8.10	7.84

*P_e_*, pool exposure rate; PARP, population attributable risk proportion.

### Sensitivity analyses

Comparison of the results of pooled ORs for the random-effects and fixed-effects models through sequential and one-by-one omission of individual studies revealed that the corresponding results were not significantly different in most of the risk factors and that the heterogeneity indicators were reduced in some conditions. However, when we removed the studies of Correa *et al*.,^33^ or Gómez Rueda and Zuleta Tobón,^13^ or Patel *et al.*,^21^ the ORs and the corresponding 95% CIs for transplant recipient changed to 1.86 (95% CI = [0.85–4.07]), 1.98 (95% CI = [0.81–4.85]), and 1.49 (95% CI = [0.86–2.61]), respectively. Similarly, when we removed the study of Correa,^33^ the ORs and the corresponding 95% CI for steroids use changed from 1.43 (95% CI = [1.04–1.96]) to 1.34 (95% CI = [0.97–1.86]). The results changed and became statistically insignificant for the transplant recipient factor and steroids use factor.

### Publication bias

The publication bias among the included studies was evaluated with the Egger's test, which had stronger statistical power to provide evidence of publication bias.^36^ No obvious asymmetry of the risk factors was observed except tracheostomy (*p* = 0.036), and the results are shown in Table 2.

## Discussion

In this meta-analysis, we aimed to identify the risk factors of CRKP infection by summarizing the results of relevant articles published so far. Since it was first identified in North Carolina in 1996, CRKP has become the most frequent CRE species found in the United States.^35^ CRKP has also been endemic in other areas worldwide, including China,^37^ Israel,^38^ a number of South America countries,^39,40^ and Europe, especially Greece and Italy.^7^ The rapid and global dissemination of CRKP has become a substantial concern in healthcare facilities. Infections with such strains are difficult to eradicate and have a limited treatment options. Therefore, determining the possibility of CRKP infection in early stage through risk factors and taking reasonable prevention could be helpful to reduce the incidence rate of CRKP infection. Although some studies on the risk factors of CRKP infection are previously available, their results are not always consistent. These differences could be due to the insufficient statistical power of individual studies with small sample sizes or the variations that exist in different selection criteria and study designs. Furthermore, no meta-analysis study exists on this topic until now. Accordingly, we performed this meta-analysis to identify the relationship between risk factors and CRKP infection.

In consideration of the defined inclusion and exclusion criteria, 3,627 participants were included in our study. Most of the eligible studies clearly manifested the total sample size, inclusion and exclusion criteria of subjects, and characteristics of participants. Furthermore, all the included studies were evaluated as high quality during the quality assessment. Thus, we concluded that the results based on existing evidence were relatively convincing. The 16 included articles mainly originated from eight nations, 4 were published in the United States,^21,23,27,31^ where the CRKP was the most prevalent strain among CRE; 5 were from Greece and Italy,^16,25,26,30,32^ the two countries with the highest percentage of CRKP in Europe,^41^ which also contributed to the vast majority of infection proportion; and the remaining were from China, Brazil, Colombia, Turkey, and Israel, where CRKP infection was geographically epidemic. Among these studies, one^27^ only focused on urinary tract infection, three^25,26,32^ focused on bloodstream infection, and the rest included patients with different infection types.

In this meta-analysis, we found that previous antibiotic use is a risk factor for CRKP infection, and the PARP of the previous antibiotic use up to 62.42% was the highest PARP of our analysis, indicating that diverse specific antibiotics have been utilized across published studies and previous antibiotic use is an important risk factor of CRKP infection. In our meta-analysis, patients exposed to main antibiotics, such as carbapenems, aminoglycosides, glycopeptides, quinolones, and anti-pseudomonal penicillins, have a higher risk of acquiring CRKP infection. These factors were determined as such because antibiotic selective pressure is the main cause of the drug-resistant strain infections. A study has suggested that the use of carbapenem antibiotics was closely related to the production of *K. pneumoniae* carbapenemase.^42^ Increased exposure to one antibiotic group boosts the effect of exposure to the other antibiotic group on CRKP infection risk.^43^ Therefore, combined use of antibiotics and longer treatment with carbapenems in large doses have increased the antibiotic selection pressure, allowing carbapenem-resistant bacteria to develop a plethora of carbapenem resistance mechanisms.^44^

Undergoing invasive procedures increases the risk of infection. However, our meta-analysis revealed that only the use of CVC, MV, tracheostomy, and parenteral nutrition exhibited statistical significance. Intubation or tracheotomy destroys the normal human body barrier, which facilitates contact between the interior of the human body and the external environment, causing opportunistic pathogens to easily invade and attach to the inner wall of the intubation where they form a biofilm cover that is difficult to eradicate; consequently, the pathogenic bacteria can enter the deep tissue of the body, increasing the chance of CRKP infection.^45^ Notably, we found a publication bias for tracheostomy among the studies using Egger's (*p* = 0.036) test, which may be caused by the inclusion of few component studies and the small sample size. Therefore, further studies about the relationship between tracheostomy and CRKP infection are needed.

Furthermore, our study found that prior hospitalization and longer LOS were risk factors for CRKP infection development. The result may be explained by the fact that patients with previous hospitalization and longer LOS before CRKP infection have an increased infection risk because of prolonged exposure to invasive devices or antibiotic use.^13^ Similarly, admission to ICU is closely associated with the occurrence of CRKP infection. Compared with the non-infected patients, those with CRKP infection have prolonged days of ICU stay. Carbapenem resistance is more common among *Klebsiella* spp. isolated from the ICU compared with non-ICU isolates patients.^46^ This finding may be because ICU patients are usually extremely vulnerable and critically ill with a prolonged hospital stay, various antibiotic types and dosages exposure, and a high number of invasive procedures performed. Furthermore, carbapenems are the mainstay empiric antibiotic therapy for severe ICU-acquired infections caused by drug-resistant Gram-negative bacteria, which may further explain the emergence of CRKP infection in the ICU.^47^

In our study, we provided statistically significant evidence for transplant recipient and steroids use, but the findings were sometimes unstable. The results changed and became statistically insignificant for the transplant recipient factor when the studies of Correa *et al.*,^33^ or Gómez Rueda and Zuleta Tobón,^13^ or Patel *et al.*^21^ were removed. In addition, the result of steroids use factor also became statistically insignificant with the removal of the study of Correa *et al.*.^33^ The results of the two sensitivity analyses could be attributed to the small sample size, which led to the overrating of the combined effect and the inversion of the conclusion.

We should also pay attention to several limitations of our study, which may affect the results. First, we only included published studies from five databases. Hence, relevant articles published in other databases and unpublished studies might have been missed. Second, we excluded some studies because of an unclear diagnosis criterion of infection or they did not distinguish the outcomes of infected patients from those of colonized patients, which led to the extremely small dataset collection in the inclusion and limited the statistical power to detect possible independent risk factors for CRKP infection. Third, significant heterogeneity was detected in some risk factors because we had strict enrollment criteria of references (only included case–control or cohort study). In addition, the inclusive studies were conducted in different countries, and some diagnostic levels and the basic condition of the eligible patients might have varied significantly. Therefore, the heterogeneity between the included studies could be high.

In conclusion, we identified a number of factors associated with CRKP infection development. These findings may provide impetus for infection control, promote rational use of available antibiotics, and provide containment of CRKP spread. Further well-designed and large randomized controlled trials and other intervention evaluation studies are needed to develop effective preventive and therapeutic protocols for CRKP infection in the future.

## References

[1] Paterson D.L. 2006 Resistance in gram-negative bacteria: enterobacteriaceae. Am. J. Med. 119:S20–S28; discussion S62–S70 10.1016/j.amjmed.2006.03.013 16735147

[2] Martirosov D.M., and Lodise T.P. 2016 Emerging trends in epidemiology and management of infections caused by carbapenem-resistant Enterobacteriaceae. Diagn. Microbiol. Infect. Dis. 85:266–275 27033631 10.1016/j.diagmicrobio.2015.10.008

[3] Falagas M.E., Tansarli G.S., Karageorgopoulos D.E., and Vardakas K.Z. 2014 Deaths attributable to carbapenem-resistant Enterobacteriaceae infections. Emerg. Infect. Dis. 20:1170–1175 24959688 10.3201/eid2007.121004 PMC4073868

[4] Bogan C., Kaye K.S., Chopra T., Hayakawa K., Pogue J.M., Lephart P.R., Bheemreddy S., Lazarovitch T., Zaidenstein R., Perez F., Bonomo R.A., and Marchaim D. 2014 Outcomes of carbapenem-resistant Enterobacteriaceae isolation: matched analysis. Am. J. Infect. Control 42:612–620 24837111 10.1016/j.ajic.2014.02.013

[5] Podschun R., and Ullmann U. 1998 *Klebsiella* spp. as nosocomial pathogens: epidemiology, taxonomy, typing methods, and pathogenicity factors. Clin. Microbiol. Rev. 11:589–603 9767057 10.1128/cmr.11.4.589 PMC88898

[6] European Centre for Disease Prevention and Control (ECDC). 2006 The European Antimicrobial Resistance Surveillance System (EARSS) Annual Report 2005. Available at https://ecdc.europa.eu/en/about-us/partnerships-and-networks/disease-and-laboratory-networks/ears-net (accessed May 12, 2017)

[7] European Centre for Disease Prevention and Control (ECDC). 2012 Antimicrobial resistance surveillance in Europe 2011. Annual Report of the European Antimicrobial Resistance Surveillance Network (EARS-Net). Available at http://ecdc.europa.eu/en/publications/_layouts/forms/Publication_DispForm.aspx?List=4f55ad51-4aed-4d32-b960-af70113dbb90&ID=719 (accessed 512, 2017)

[8] Borer A., Saidel-Odes L., Riesenberg K., Eskira S., Peled N., Nativ R., Schlaeffer F., and Sherf M. 2009 Attributable mortality rate for carbapenem-resistant *Klebsiella pneumoniae* bacteremia. Infect. Control Hosp. Epidemiol. 30:972–976 19712030 10.1086/605922

[9] Bratu S., Landman D., Haag R., Recco R., Eramo A., Alam M., and Quale J. 2005 Rapid spread of carbapenem-resistant *Klebsiella pneumoniae* in New York City: a new threat to our antibiotic armamentarium. Arch. Intern. Med. 165:1430–1435 15983294 10.1001/archinte.165.12.1430

[10] Neuner E.A., Yeh J.Y., Hall G.S., Sekeres J., Endimiani A., Bonomo R.A., Shrestha N.K., Fraser T.G., and van Duin D. 2011 Treatment and outcomes in carbapenem-resistant *Klebsiella pneumoniae* bloodstream infections. Diagn. Microbiol. Infect. Dis. 69:357–362 21396529 10.1016/j.diagmicrobio.2010.10.013 PMC3058153

[11] Gupta N., Limbago B.M., Patel J.B., and Kallen A.J. 2011 Carbapenem resistant Enterobacteriaceae: epidemiology and prevention. Clin. Infect. Dis. 53:60–67 21653305 10.1093/cid/cir202

[12] Kwak Y.G., Choi S.-H., Choo E.J., Chung J.-W., Jeong J.-Y., Kim N.J., Woo J.-H., Ryu J., and Kim Y.S. 2005 Risk factors for the acquisition of carbapenem-resistant *Klebsiella pneumoniae* among hospitalized patients. Microb. Drug Resist. 11:165–169 15910232 10.1089/mdr.2005.11.165

[13] Gómez Rueda V., and Zuleta Tobón J.J. 2014 Risk factors for infection with carbapenem-resistant *Klebsiella pneumoniae*: a case-case-control study. Colomb. Méd. 45:54–60 25100889 PMC4123582

[14] Wu D., Cai J., and Liu J. 2011 Risk factors for the acquisition of nosocomial infection with carbapenem-resistant *Klebsiella pneumoniae*. South Med. J. 104:106–110 21258230 10.1097/SMJ.0b013e318206063d

[15] Akgul F., Bozkurt I., Sunbul M., Esen S., and Leblebicioglu H. 2016 Risk factors and mortality in the Carbapenem-resistant *Klebsiella pneumoniae* infection: case control study. Pathog. Glob. Health. 110:321–325 27903130 10.1080/20477724.2016.1254976 PMC5189867

[16] Falagas M.E., Rafailidis P.I., Kofteridis D., Virtzili S., Chelvatzoglou F.C., Papaioannou V., Maraki S., Samonis G., and Michalopoulos A. 2007 Risk factors of carbapenem-resistant *Klebsiella pneumoniae* infections: a matched case—control study. J. Antimicrob. Chemother. 60:1124–1130 17884829 10.1093/jac/dkm356

[17] Delahaye F., Landrivon G., Ecochard R., and Colin C. 1991 Meta-analysis. Health Policy 19:185–196 10115991 10.1016/0168-8510(91)90007-k

[18] Moher D., Liberati A., Tetzlaff J., and Altman D.G. 2009 Preferred reporting items for systematic reviews and meta-analyses: the PRISMA statement. J. Clin. Epidemiol. 62:1006–1012 19631508 10.1016/j.jclinepi.2009.06.005

[19] Wells G.A., Shea B., O'Connell D., Peterson J., Welch V., Losos M., and Tugwell P. The Newcastle-Ottawa Scale (NOS) for assessing the quality of nonrandomised studies in meta-analysis. Available at http://www.ohri.ca/programs/clinical_epidemiology/oxford.asp (accessed 512, 2017)

[20] Ownby R.L., Crocco E., Acevedo A., John V., and Loewenstein D. 2006 Depression and risk for Alzheimer disease: systematic review, meta-analysis, and metaregression analysis. Arch. Gen. Psychiatry. 63:530–538 16651510 10.1001/archpsyc.63.5.530 PMC3530614

[21] Patel G., Huprikar S., Factor S.H., Jenkins S.G., and Calfee D.P. 2008 Outcomes of carbapenem-resistant *Klebsiella pneumoniae* infection and the impact of antimicrobial and adjunctive therapies. Infect. Control Hosp. Epidemiol. 29:1099–1106 18973455 10.1086/592412

[22] Borer A., Saidel-Odes L., Eskira S., Nativ R., Riesenberg K., Livshiz-Riven I., Schlaeffer F., Sherf M., and Peled N. 2012 Risk factors for developing clinical infection with carbapenem-resistant *Klebsiella pneumoniae* in hospital patients initially only colonized with carbapenem-resistant *K pneumoniae*. Am. J. Infect. Control. 40:421–425 21906844 10.1016/j.ajic.2011.05.022

[23] Simkins J., Muggia V., Cohen H.W., and Minamoto G.Y. 2014 Carbapenem-resistant *Klebsiella pneumoniae* infections in kidney transplant recipients: a case-control study. Transpl. Infect. Dis. 16:775–782 25092500 10.1111/tid.12276

[24] Hu Y., Ping Y., Li L., Xu H., Yan X., and Dai H. 2016 A retrospective study of risk factors for carbapenem-resistant *Klebsiella pneumoniae* acquisition among ICU patients. J. Infect. Dev. Ctries. 10:208–213 27031451 10.3855/jidc.6697

[25] Giannella M., Trecarichi E.M., De Rosa F.G., Del Bono V., Bassetti M., Lewis R.E., Losito A.R., Corcione S., Saffioti C., Bartoletti M., Maiuro G., Cardellino C.S., Tedeschi S., Cauda R., Viscoli C., Viale P., and Tumbarello M. 2014 Risk factors for carbapenem-resistant *Klebsiella pneumoniae* bloodstream infection among rectal carriers: a prospective observational multicentre study. Clin. Microbiol. Infec. 20:1357–1362 24980276 10.1111/1469-0691.12747

[26] Mouloudi E., Massa E., Papadopoulos S., Iosifidis E., Roilides I., Theodoridou T., Piperidou M., Orphanou A., Passakiotou M., Imvrios G., Fouzas I., Papanikolaou V., and Gritsi-Gerogianni N. 2014 Bloodstream infections caused by carbapenemase-producing *Klebsiella pneumoniae* among intensive care unit patients after orthotopic liver transplantation: risk factors for infection and impact of resistance on outcomes. Transplant. Proc. 46:3216–3218 25420863 10.1016/j.transproceed.2014.09.159

[27] Brizendine K.D., Richter S.S., Cober E.D., and Van Duin D. 2015 Carbapenem-resistant *Klebsiella pneumoniae* urinary tract infection following solid organ transplantation. Antimicrob. Agents Chemother. 59:553–557 25385105 10.1128/AAC.04284-14 PMC4291398

[28] Candevir Ulu A., Kurtaran B., Inal A.S., Kömür S., Kibar F., Yapıcı Çiçekdemir H., Bozkurt S., Gürel D., Kılıç F., Yaman A., Aksu H.S.Z., and Taşova Y. 2015 Risk factors of carbapenem-resistant *Klebsiella pneumoniae* infection: a serious threat in ICUs. Med. Sci. Monit. 21:219–224 25595166 10.12659/MSM.892516 PMC4304439

[29] Freire M.P., Abdala E., Moura M.L., de Paula F.J., Spadão F., Caiaffa-Filho H.H., David-Neto E., Nahas W.C., and Pierrotti L.C. 2015 Risk factors and outcome of infections with *Klebsiella pneumoniae* carbapenemase-producing *K. pneumoniae* in kidney transplant recipients. Infection 43:315–323 25690848 10.1007/s15010-015-0743-4

[30] Giannella M., Bartoletti M., Morelli M.C., Tedeschi S., Cristini F., Tumietto F., Pasqualini E., Danese I., Campoli C., Lauria N.D., Faenza S., Ercolani G., Lewis R., Pinna A.D., and Viale P. 2015 Risk factors for infection with carbapenem-resistant *Klebsiella pneumoniae* after liver transplantation: the importance of pre- and posttransplant colonization. Am. J. Transplant. 15:1708–1715 25754742 10.1111/ajt.13136

[31] Ny P., Nieberg P., and Wong-Beringer A. 2015 Impact of carbapenem resistance on epidemiology and outcomes of nonbacteremic *Klebsiella pneumoniae* infections. Am. J. Infect. Control 43:1076–1080 26190386 10.1016/j.ajic.2015.06.008

[32] Vardakas K.Z., Matthaiou D.K., Falagas M.E., Antypa E., Koteli A., and Antoniadou E. 2015 Characteristics, risk factors and outcomes of carbapenem-resistant *Klebsiella pneumoniae* infections in the intensive care unit. J. Infect. 70:592–599 25447713 10.1016/j.jinf.2014.11.003

[33] Correa L., Martino M.D., Siqueira I., Pasternak J., Gales A.C., Silva C.V., Camargo T.Z., Scherer P.F., and Marra A.R. 2013 A hospital-based matched case-control study to identify clinical outcome and risk factors associated with carbapenem-resistant *Klebsiella pneumoniae* infection. BMC Infect. Dis. 13:80 23398691 10.1186/1471-2334-13-80 PMC3574843

[34] Higgins J.P., and Thompson S.G. 2002 Quantifying heterogeneity in a meta-analysis. Stat. Med. 21:1539–1558 12111919 10.1002/sim.1186

[35] Centers for Disease Control and Prevention (CDC). 2009 Guidance for control of infections with carbapenem-resistant or carbapenemase-producing Enterobacteriaceae in acute care facilities. MMWR Morb. Mortal. Wkly Rep. 58:256–260 19300408

[36] Hayashino Y., Noguchi Y., and Fukui T. 2005 Systematic evaluation and comparison of statistical tests for publication bias. J. Epidemiol. 15:235–243 16276033 10.2188/jea.15.235 PMC7904376

[37] Shen P., Wei Z., Jiang Y., Du X., Ji S., Yu Y., and Li L. 2009 Novel genetic environment of the carbapenem-hydrolyzing beta-lactamase KPC-2 among Enterobacteriaceae in China. Antimicrob. Agents Chemother. 53:4333–4338 19620332 10.1128/AAC.00260-09 PMC2764158

[38] Navon-Venezia S., Leavitt A., Schwaber M.J., Rasheed J.K., Srinivasan A., Patel J.B., and Carmeli Y. 2009 First report on a hyperepidemic clone of KPC-3-producing *Klebsiella pneumoniae* in Israel genetically related to a strain causing outbreaks in the United States. Antimicrob. Agents Chemother. 53:818–820 19029323 10.1128/AAC.00987-08 PMC2630632

[39] Nordmann P., Cuzon G., and Naas T. 2009 The real threat of *Klebsiella neumoniae* carbapenemase-producing bacteria. Lancet Infect. Dis. 9:228–236 19324295 10.1016/S1473-3099(09)70054-4

[40] Pavez M., Mamizuka E.M., and Lincopan N. 2009 Early dissemination of KPC-2-producing *Klebsiella pneumoniae* strains in Brazil. Antimicrob. Agents Chemother. 53:2702 19332672 10.1128/AAC.00089-09 PMC2687248

[41] European Centre for Disease Prevention and Control (ECDC). 2015 Antimicrobial resistance surveillance in Europe 2014. Annual Report of the European Antimicrobial Resistance Surveillance Network (EARS-Net). Available at https://ecdc.europa.eu/en/publications-data/antimicrobial-resistance-surveillance-europe-2014 (accessed 723, 2017)

[42] del Mar Tomas M., Cartelle M., Pertega S., Beceiro A., Llinares P., Canle D., Molina F., Villanueva R., Cisneros J.M., and Bou G. 2005 Hospital outbreak caused by a carbapenem-resistant strain of *Acinetobacter baumannii*: patient prognosis and risk-factors for colonisation and infection. Clin. Microbiol. Infect. 11:540–546 15966971 10.1111/j.1469-0691.2005.01184.x

[43] Kritsotakis E.I., Tsioutis C., Roumbelaki M., Christidou A., and Gikas A. 2011 Antibiotic use and the risk of carbapenem-resistant extended-spectrum-{beta}-lactamase-producing *Klebsiella pneumoniae* infection in hospitalized patients: results of a double case-control study. J. Antimicrob. Chemother. 66:1383–1391 21454344 10.1093/jac/dkr116

[44] Peleg A.Y., and Hooper D.C. 2010 Hospital-acquired infections due to gram-negative bacteria. N. Engl. J. Med. 362:1804–1813 20463340 10.1056/NEJMra0904124 PMC3107499

[45] Girmenia C., Rossolini G.M., Piciocchi A., Bertaina A., Pisapia G., Pastore D., Sica S., Severino A., Cudillo L., Ciceri F., Scime R., Lombardini L., Viscoli C., and Rambaldi A. 2015 Infections by carbapenem-resistant *Klebsiella pneumoniae* in SCT recipients: a nationwide retrospective survey from Italy. Bone Marrow Transplant. 50:282–288 25310302 10.1038/bmt.2014.231

[46] Sader H.S., Farrell D.J., Flamm R.K., and Jones R.N. 2014 Antimicrobial susceptibility of Gram-negative organisms isolated from patients hospitalized in intensive care units in United States and European hospitals (2009–2011). Diagn. Microbiol. Infect. Dis. 78:443–448 24492025 10.1016/j.diagmicrobio.2013.11.025

[47] MacVane S.H. 2017 Antimicrobial resistance in the intensive care unit: a focus on gram-negative bacterial infections. J. Intensive Care Med. 32:25–37 26772199 10.1177/0885066615619895

